# Unicompartmental knee arthroplasty for tricompartment osteoarthritis in octogenarians

**DOI:** 10.4103/0019-5413.54970

**Published:** 2009

**Authors:** SKS Marya, Rajiv Thukral

**Affiliations:** Max Institute of Orthopedics and Joint Replacement, Max Super-Specialty Hospital, 1, Press Enclave Road, Saket, New Delhi – 110017, India

**Keywords:** Octogenarian, tricompartmental arthritis, unicompartmental knee arthroplasty

## Abstract

**Background:**

Unicompartmental knee arthroplasty (UKA) is specifically indicated in isolated unicompartmental arthritis with competent ligaments. Recent series of UKA for unicompartmental arthritis have shown good function, persistence of pain relief, and nearly 90% survivorship at 15 years, even in knees that would perhaps not be considered good indications for UKA today. The perioperative morbidity of UKA is less than total knee arthroplasty. We present our series of 19 octogenarians with tricompartment osteoarthritis (predominant medial compartment involvement) treated with UKA as definitive surgery.

**Materials and Methods:**

We performed UKA on 29 knees (19 patients) average 83 years (79-94 years) of either sex from Jan 2002 to Dec 2006. All the patients had tricompartment knee osteoarthritis (with predominant medial and some patellofemoral compartment involvement).

**Results:**

The results were evaluated using the Knee Society scores and visual analogue score over an average 48-month follow-up (range, 24 to 81 months). Barring one (medial femoral condyle fracture detected on postoperative radiography), all patients achieved promised levels of satisfaction.

**Discussion:**

UKA for tricompartment knee arthritis in the young active patient entails risk of dissatisfaction and failure. We present UKA in select ‘very elderly’ patients with tricompartment osteoarthritis (with predominant unicompartment involvement).

## INTRODUCTION

Unicompartmental arthritis (medial, lateral or patellofemoral) is the result of mechanical malalignment which merits correction to prevent its deterioration to tricompartment arthritis.^1^–^2^ Multiple intrinsic patient influences (genetics, bone stock, compliance) and extrinsic factors (gender, weight, absence of inflammatory arthritis, osteopenia, osteoporosis, preoperative deformity, revision osteotomy surgery, etc.) need evaluation as possible etiologic factors.^1^

Surgical treatment of advanced unicompartmental knee arthritis (with functionally intact ligaments) has shown limited success with distal femoral or proximal tibial osteotomy^1^–^2^ (lateral closed wedge, medial open wedge, and/or modifications thereof). An alternative in select patients is unicompartmental knee arthroplasty (UKA). UKA recreates joint space in the compartment that has collapsed. The cruciate ligaments and extensor mechanism are left intact,^3^ providing for near-normal mechanics and kinematics.^3^–^4^

Initial results using older prosthetic designs reportedly failed either due to nonadherence to strict indication criteria, or to progression of osteoarthritis in the other compartments.^5^–^6^ Recent series of UKA for unicompartmental arthritis have shown good function, persistence of pain relief, and nearly 90% survivorship^7^–^9^ at 15 years, even in knees that would perhaps not be considered good indications for UKA today.^8^–^9^ However, the procedure needs to be technically exact. Some eminent researchers have gone on to suggest that UKA is a temporizing procedure,^10^–^11^ adding roughly a decade to the prosthetic life span. Reviews from the Swedish and Finnish registry have confirmed that good long term results following UKA are related to the number of UKAs performed.^11^ With eventual failure of the UKA, prognosis after revision to a TKA is nearly the same as that following primary TKA.^12^

We present early results in 19 octogenarians (29 knees) with tricompartment arthritis (predominant medial and patellofemoral compartment involvement) treated with UKA and present the procedure as a definitive surgical option for a select patient group, The aim was to provide improved knee function and pain while reducing perioperative morbidity (as seen with TKA) in this tricompartment knee arthritis patient group.

## MATERIALS AND METHODS

**Figure 1:**
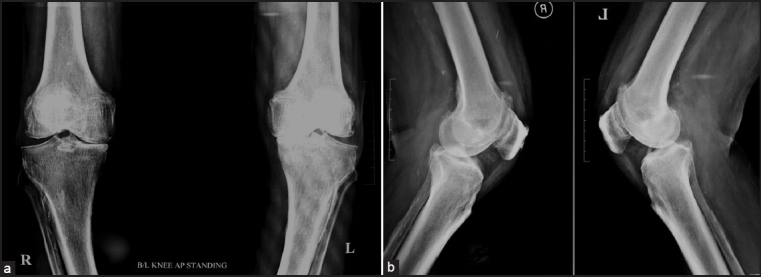
Preoperative anteroposterior (a) and lateral (b) radiographs of both knee of 81-year old patient showing evidence of tri-compartment arthritis

Nineteen consecutive octogenarian patients (29 knees) were operated upon by the senior author over a period of four years (Jan 2002 to Dec 2006). Average age of the patient was 83 years (range, 79 to 94 years). There were three females and 16 males. Selection criteria included advanced age (over 79 years) and painful tricompartment knee osteoarthritis [Figure 1a and b], with predominant medial tibiofemoral compartment with/without patellofemoral compartment involvement. All patients had preoperative anterior and medial knee pain, with mild or no lateral joint pain. All had varus deformity (0 to 15°), with an average range of motion (ROM) of 100° (80 to 115°). Knee Society scores for pain and function were recorded [Table 1]. Patients with inflammatory arthritis (rheumatoid arthritis, seronegative arthritis, gouty arthritis, etc.), anterior cruciate ligament (ACL) incompetence^8^ and advanced lateral tibiofemoral or patellofemoral compartment arthritis were excluded from our series.^5^ High body mass index (BMI) was not an exclusion criterion. All patients were preoperatively explained the possible need to proceed to TKA surgery (and consented as such) if evidence of advanced tricompartment arthritis was found intraoperatively.^13^ All patients had changes in the lateral and patellofemoral compartment (Allgower's grade II – III) but no additional procedure (patellaplasty or lateral condylar shaving) was done.

Ten patients underwent simultaneous bilateral UKA surgery, while the remaining nine had unilateral replacement. All underwent medial compartment UKA using the Allegretto™ system (Zimmer, Warsaw, IN, USA). The average duration of each surgery was 43 minutes (range, 33 to 58 minutes). A tourniquet was used in all cases, and the average postoperative blood loss (drain) was 150ml (range, 100 to 220ml). Combined spinal epidural anesthesia was used in all patients, with epidural analgesia continued till the second postoperative day. Routine DVT chemoprophylaxis supplemented with graduated compression stockings was used till discharge. Average length of hospital stay was four (unilateral UKA) and six days (bilateral UKA) respectively.

Postoperatively, patients were made to stand and walk full weight bearing with a walking stick on the first postoperative day. Knee bending progressed from 30° on the third day to 90° at two weeks. Patients were evaluated preoperatively at three months, six months, 12 months and yearly thereafter, clinically and radiographically. Knee Society scores (clinical and function) and satisfaction, measured using the visual analogue score (VAS), were used as outcome measures, with special emphasis on return to activities of daily living [Table 1].

## RESULTS

**Figure 2:**
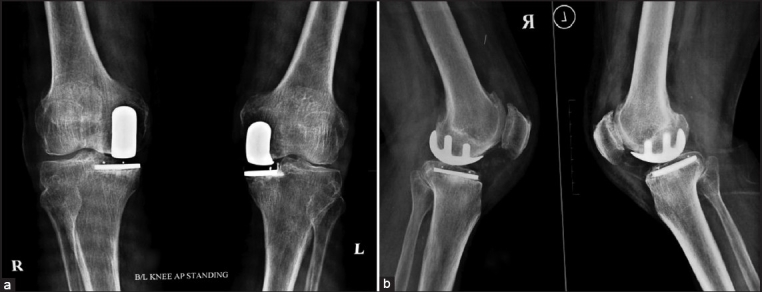
Anteroposterior (a) and lateral (b) radiographs of same patient 48 months post-bilateral UKA

**Figure 3:**
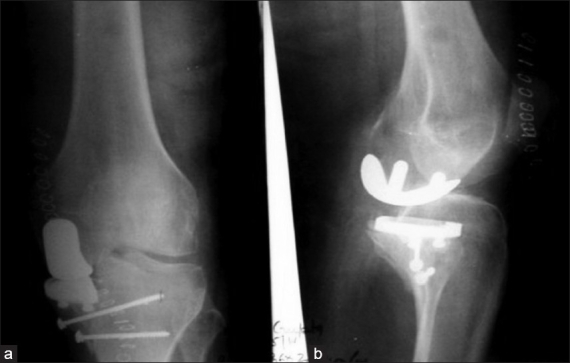
Immediate post-fixation anteroposterior view (a) and lateral view (b) radiographs of patient who had an undiagnosed medial tibial condyle fracture

At final follow-up (average, 48 months, range 24 to 82 months), all (but one) patients were pain-free, walking independently, and had an average knee range of motion (ROM) of 115° (range, 100 to 125°) [Figure 2a and b]. One patient had persistent pain in the knee immediately postoperatively, and was diagnosed to have a missed intraoperative undisplaced fracture of the medial tibial plateau. He was treated with percutaneous screw fixation on the 10th post-operative day [Figure 3], with no bearing on the end result at six months. One patient developed superficial infection along the wound margins which settled with antibiotics, with no effect on the subsequent rehabilitation or end result. In this short term study, we observed no radiological signs of lysis or loosening. No patient had any of the complications associated with TKA, viz. deep vein thrombosis (DVT), pulmonary embolism (PE), restricted flexion/extension, or mid-flexion instability,

We had excellent results in the short term studied. All but one of our patients (95%) showed improvement in their pain and function scores, with special regard to improved mobility, pain reduction, and the resumption of activities of daily living. This particular patient (E07) [Table 1] had poor pain (72) and function scores (70) even at 48 months follow-up. This was probably due to improper patient selection (undiagnosed seronegative arthritis) with subsequent persistence of the inflammatory etiology. He underwent conversion to bilateral TKR at a date after the study period ended. Mean Knee Society clinical scores improved from 49 (range, 32 to 70) preoperatively, to 84 (range, 72 to 100) postoperatively. Similarly, mean Knee Society function scores also improved from 30 (range, 0 to 50) to 80 (range, 55 to 90). Satisfaction with the procedure showed the most dramatic improvement in scores. VAS improved from a mean preoperative of three (range, 0 to 6) to 9 (8 to 10) at six months postoperatively, with a final value of eight (range, 7 to 10) at final follow-up of 48 months [Table 1].

**Table 1:** Clinical details of patient demographics, preoperative and follow-up scores

Pt. details			Procedure (uni/bilateral)	Pre-op KSS and VAS			Total duration of follow up (months)	Post-op KSS and VAS (last follow up)			Complications/major events during early postop period
					
ID	Age (yrs)	Sex		Clinical score	Function score	VAS score		Clinical score	Function score	VAS score	
E01	79	M	Unilateral	70	45	6	81	100	85	9	-
E02	87	M	Unilateral	61	40	4	79	88	80	8	Early # femoral condyle, fixed 10 days post-op
E03	83	M	Unilateral	61	40	4	75	92	80	8	-
E04	80	M	Bilateral	46	25	3	69	85	80	7	-
E05	88	M	Unilateral	39	35	2	61	92	85	9	-
E06	94	M	Unilateral	61	40	4	58	88	75	8	-
E07	80	M	Bilateral	32	15	1	55	72	70	7	-
E08	81	M	Bilateral	41	20	3	49	85	80	8	-
E09	79	M	Bilateral	46	20	3	46	82	80	8	-
E10	82	F	Bilateral	32	10	1	44	72	55	7	Superficial Left knee wound infection
E11	89	M	Unilateral	61	40	4	41	77	80	8	-
E12	79	M	Bilateral	46	50	5	35	92	90	10	-
E13	86	F	Unilateral	46	30	3	34	82	80	8	-
E14	81	M	Bilateral	61	20	2	33	85	80	9	-
E15	82	M	Bilateral	32	0	0	33	72	75	8	-
E16	81	M	Bilateral	41	20	2	32	75	80	8	-
E17	84	M	Unilateral	61	50	4	29	85	90	8	-
E18	81	M	Bilateral	39	15	1	26	85	80	9	-
E19	82	F	Unilateral	61	50	3	24	92	90	10	-

ID = Identity Number, Pre-op = Preoperative, Post-op = Postoperative, KSS = Knee society score, # = Fracture, VAS = Visual analogue score

## DISCUSSION

Advanced tricompartment knee osteoarthritis in the elderly has been successfully managed with TKA with many reported results of 90 to 95% survivorship at up to 20 years follow up,^14^–^15^ UKA has been reserved for nonobese less active elderly patients with advanced non-inflammatory unicompartmental arthritis^1^,^5^ and within these indications, has demonstrated good success, both in terms of function and longevity.^8^–^9^,^16^ Series with case-matched young patients (age 55 to 65 years) have repeatedly stressed the inability of UKA to provide longer term reliable results when compared with TKA.^17^ Success has been specifically seen with stringent inclusion and exclusion criteria,^1^,^5^ but patients with marginal other compartment involvement,^13^,^18^ younger age^19^ and ACL incompetence^3^,^8^ have shown matched good results.^19^ Berger *et al*.^20^ have shown that progression of arthritis in the lateral or patellofemoral compartments is not likely to affect long term prognosis of UKA. Price *et al*,^17^ demonstrated that although UKA has been stated to be unsuitable for highly active younger patients, up to 91% patients, aged 50 or more, can have their UKAs last 10 years or more.

Advanced unicompartmental knee arthritis, after sufficient trial of conservative treatment, has been treated by arthroscopic procedures^21^ or different osteotomies^2^,^22^ over time. However, results following these procedures have been short-lived.^2^,^21^ Although some surgeons have used tibial osteotomies to provide relief from pain for up to 10 years, there is a steep decline in joint function in these patients, thereafter, due to accelerated degeneration necessitating TKA.^22^–^23^ Revision from a high tibial osteotomy to TKA (for secondary tricompartment arthritis) has been fraught with technical difficulties and poor mid- to long-term results than those with primary TKA, both clinically and radiographically.^22^–^23^

UKA and TKA have been offered by many surgeons for unicompartmental arthritis, with reliable success and longevity,^2^,^8^–^9^,^14^–^16^ demonstrated by its more widespread prevalence today. Introduced in 1972, the UKA has undergone modifications and alterations in implant design, kinematics and fixation method, and surgical techniques.^24^–^25^ Initial excellent results at 8-10 years have given way to unacceptably high failure^5^–^6^ at 15 years. This has been attributed to implant-related (problems with design, material, fixation method, and stability), patient-related (inappropriate indication, presence of tri-compartment arthritis, excessively active lifestyle, etc.) or surgeon-related (improper technique, partial injury to cruciate ligaments or tibiofemoral articular cartilage, failure to treat the underlying pathology producing the unicompartmental arthritis, etc) issues.^5^,^7^,^26^ Complications seen after UKA have included infection, spin out of mobile meniscus, dislocation, tibial plateau fractures, femoral condyle necrosis, implant loosening and osteolysis.^5^–^6^,^12^,^15^,^19^ Most of these are now known to be preventable, as they depend on appropriate indication and meticulous surgical technique.^8^–^9^,^25^

Advantages cited in favor of UKA vis-à-vis TKA include early and complete knee range of motion (maintenance of the suprapatellar space and infrapatellar fat pad), early recuperation and rehabilitation (smaller incision and minimal tissue trauma), preservation of the normal knee biology (bone stock, kinematics, proprioception and function), and lower morbidity (lesser blood loss, shorter hospital stay, and fewer complications).^27^–^28^ Another advantage frequently stated is the ease of conversion to a TKA at any stage (during surgery, or in the follow up), and eventual outcomes matching primary TKA for arthritis.^12^,^28^ Further, as no medullary canal is breached, risk of fat embolism as well as deep vein thrombosis is less.^29^ Survival data from the Norwegian Arthroplasty register has demonstrated best cost-effectiveness with UKA.^30^

The fact that octogenarians have a sedentary life style and are more prone to morbidity and mortality following knee replacement surgery (UKA or TKA) does not imply that they do not deserve such a procedure when otherwise indicated.^31^–^32^ There is little doubt that TKA surgery has the best track record, in terms of pain relief, function and longevity today, as has been seen time and again by many reports worldwide.^14^–^16^ However, TKA in this group of fragile very elderly population can result in morbidity, delayed rehabilitation and mortality (with coexistent multiple medical morbidity). The stress and demands posed by the TKA procedure may cause sudden deterioration of health and pose significant morbidity risk^27^–^28^,^33^ (higher post-operative complications after TKA than those after UKA).

UKA in these very elderly patients is potentially ideal as it affords certain unique advantages not provided by either osteotomy or TKA.^34^ Due to lower physiological activity level and lowered life-expectancy, fears of revision surgery (and associated risks and complications) are minimized. The morbidity and complications associated with UKA are relatively low, recovery is quick, and results in the form of pain relief and rehabilitation simpler, providing high satisfaction levels. Return to activities of daily living, which is perhaps most crucial in this age group of patients, is very rapid.^24^,^33^–^34^ Further, they pose very low demand on the prosthetic knee, with possibility of outliving the prosthetic knee.^33^–^34^

Early results reported by us match those seen with similar patient cohorts reported in literature.^27^,^29^,^32^–^35^ Limitations of our study include the small size of our study group, difficulty in objectively assessing significant lateral and patellofemoral arthritis, and non-blinding of the patients. Early results indicate that the UKA is viable option for the very elderly with tri-compartment arthritis. Studies comparing UKA and TKA in very elderly low-demand patients have proven that UKA can provide similar results, with reduced morbidity.^27^–^28^,^30^,^35^ The one complication encountered (medial tibial plateau fracture) can partly be attributed to osteoporotic metaphyseal bone and partly to a possible stress fracture during the procedure.^35^ Prevention, early recognition, and appropriate management of such fractures is possible only with a high index of suspicion, and this can either be rectified intra-operatively, with additional screw fixation, or postoperatively, with secondary fixation.

With precise patient selection and appropriate surgical technique,^3^,^5^,^13^,^18^ UKA may prove to be the definitive surgery in a properly selected very elderly patient with tri-compartment knee osteoarthritis (predominant unicompartment involvement).^34^ The lessons learnt are - to choose patients appropriately, avoid intra and postoperative complications by anticipation and early treatment. To conclude, UKA for tricompartment knee arthritis is an option in the very elderly. Patients with inflammatory etiology (including seronegative arthritis) must be carefully looked for and excluded. Care must be taken to recognize and prevent intra and postoperative stress fractures. Longer term follow-up is needed.
